# Does high-intensity laser therapy speed return to primary function in horses with suspensory ligament desmopathy?

**DOI:** 10.18849/ve.v8i1.564

**Published:** 2023-01-12

**Authors:** James Rushing+

**Keywords:** HORSE, SUSPENSORY LIGAMENT, DESMOPATHY, LASER, HIGH-INTENSITY LASER THERAPY, HILT, HIGH-POWER LASER THERAP

## Abstract

**PICO question:**

In horses with a suspensory ligament branch injury, does high-intensity laser therapy (energy output greater than 500 mW) combined with conservative management, compared to conservative management alone, result in a faster return to primary function?

**Clinical bottom line:**

**Category of research:**

Treatment.

**Number and type of study designs reviewed:**

The number and type of study designs that were critically appraised were two peer-reviewed studies: a non-randomised controlled clinical trial and a randomised controlled experimental study.

**Strength of evidence:**

Weak.

**Outcomes reported:**

The outcomes reported are summarised as follows: the two appraised studies report positive effects of high-intensity laser therapy (HILT) on equine tendon and ligament injuries as measured by pain to palpation, lameness, swelling and ultrasonographic and magnetic resonance imaging (MRI) evaluation of HILT-treated lesions.

**Conclusion:**

In view of the strength of evidence and the outcomes from the studies the following conclusion is made: the two appraised studies provide only weak evidence to show that horses treated with high-intensity laser therapy (HILT) and conservative management return to primary function sooner than horses treated with conservative management alone. Issues of design, methodology, statistical analysis and reporting reduce the reliability and external validity of these studies.

## How to apply this evidence in practice

The application of evidence into practice should take into account multiple factors, not limited to: individual clinical expertise, patient’s circumstances and owners’ values, country, location or clinic where you work, the individual case in front of you, the availability of therapies and resources.

Knowledge Summaries are a resource to help reinforce or inform decision making. They do not override the responsibility or judgement of the practitioner to do what is best for the animal in their care.

## Clinical scenario

Your client competes in amateur-level show jumping with her Trakehner (warmblood) gelding, aged 12 years. Your clinical examination of the horse indicates an acute injury to the medial branch of the right forelimb suspensory ligament, confirmed by ultrasound. You advise a course of conservative treatment to include rest, icing, cold-water hosing and controlled exercise. That evening, your client sends you an internet article on HILT and asks if that could accelerate the timeline for her horse's return to competition. Before responding, you want to know if evidence exists to support the use of HILT to treat equine desmopathy and how it compares to conservative management with the goal of returning a horse to competition.

**List of abbreviations:** 

AT	After HILT treatment
BT	Before HILT treatment
CSA	Cross-sectional area
DDFT	Deep digital flexor tendon
DT	During HILT treatment
HILT	High-intensity laser therapy
IQR	Interquartile range
MLMM	Multivariate linear mixed model
MRI	Magnetic resonance imaging
PICO	Patient/Population, Intervention, Comparison, Outcome
SDFT	Superficial digital flexor tendon
SL	Suspensory ligament branch
SLB	Suspensory ligament branch

## The evidence

There is little evidence that high-intensity laser therapy (HILT) is effective in treating soft tissue injuries in horses. Only two peer-reviewed controlled studies address the PICO question: a non-randomised controlled clinical trial (Zielińska et al., 2020) and a randomised controlled experimental study (Pluim et al., 2020). Two PICO-relevant studies were excluded from appraisal due to a lack of control groups: a retrospective case series examining HILT treatment of tendon / ligament injuries and the return to function of 150 sport horses (Pluim et al., 2018) and a case report describing HILT treatment of ligament injuries and the return to function of two horses (Quiney et al., 2020). Other excluded papers were a conference paper reporting HILT treatment of equine tendinopathy / desmopathy in clinical and experimental settings (Fortuna et al., 2002), an equine orthopaedic application of HILT (Zielińska et al., 2015), an examination of the effects of HILT on the surface temperature of equine skin (Zielińska et al., 2021), and a potentially PICO-relevant paper with insufficient methodological and reporting detail to appraise (Jaafar et al., 2021). Also excluded from review was Pluim et al. (2022), which examined histological properties of tissues from Pluim et al. (2020), supporting the conclusions of the earlier study.

Neither appraised paper (Pluim et al., 2020; and Zielińska et al., 2020) considers the return to function of HILT-treated horses and provides only low-quality evidence to answer the PICO question; the quality of evidence is reduced by issues of study design, methodology, and data analysis and reporting.

## Summary of the evidence

**Pluim et al. (2020):** 

Population	Horses recruited to a veterinary hospital (method not reported). Inclusion criteria: healthy horses without lameness and normal results from ultrasound examination of the suspensory ligament branches of all four limbs.
Sample Size	12 warmblood horses. Group characteristics: Five geldings, seven mares. Age: 4–12 years (mean and standard deviation not reported).
Intervention details	Surgical protocol: 48 lesions of the lateral branch of the suspensory ligament were surgically induced under general anaesthesia (one lesion in each limb of each horse). Phenylbutazone administered for 5 days post-surgery (dosage not reported). Treatment and control groups each comprised of 24 lesions. For each of the 12 horses, the right or left diagonal limb was randomly assigned to the treatment group; the other diagonal limbs assigned to the control group. The horses were equally divided into a short-term post-treatment evaluation group (4 weeks) and a long-term evaluation group (6 months). The horses in both groups were euthanised at the end of the evaluation period. Lameness evaluations were performed by a veterinarian and a motion analysis system (device not reported) weekly for four weeks, then monthly. General clinical signs and lesion heat, swelling and pain were recorded daily for each horse. High-intensity laser therapy (HILT): Laser device: Touch Life Rehab prototype with custom software, maximum output 15 W, four simultaneous wavelengths between 635 nm and 980 nm. Each horse was treated with HILT 1x daily for 20 minutes commencing day one post-surgery for 4 weeks. The laser handpiece was held perpendicular to the skin at a distance of 0.5 cm. Controlled exercise protocol: Each horse was lunged daily at trot (10 minutes increasing to 30 minutes, direction change every 5 minutes) commencing day one post-surgery for 1 week or until an obvious lameness occurred; this was done to stimulate an inflammatory response in the surgically induced lesions. Short-term group: 20 minutes hand-walk daily on hard surface for weeks 2–4. Long-term group: 20 minutes hand-walk daily on hard surface from week 2 to month 3. Trot (2–20 minutes) added in months 3–6. Canter (2–4 minutes) added in months 5 and 6. Ultrasound and colour Doppler: Examination and evaluation by the same veterinarian. Video recording and images evaluated by a second, blinded veterinarian. A total of ten ultrasound and Doppler evaluations were performed (admission, weeks 1–4 and months 2–6. Magnetic resonance imaging (MRI): MRI was performed on cadaver limbs after the horses were euthanised (short-term group at 4 weeks; long-term group at 6 months). MRI scoring was performed by three blinded evaluators, each repeating the measurements three times. Statistical analysis (treatment vs control): Independent t-test used to compare lesion transverse size and ligament transverse size. Multivariate linear mixed model (MLMM) used to compare lesion cross-sectional area (CSA) and circumference. MLMM with post-hoc Scheffé test used to compare echogenicity. Logistic regression used to compare binary Doppler signal. MLMM used to compare changes in lesion CSA, circumference and transverse size between week 1 and week 4 and week 1 and month 6. MLMM used to compare MRI measurement of CSA and mean lesion signal. Details of the MLMM and binary logistic models were not provided. Ethics approval: Ethical commission of the University of Ghent, Belgium.
Study design	Prospective, randomised, within-subject controlled experimental trial.
Outcome studied	General clinical signs including lesion heat, swelling and pain recorded daily for each horse. Subjective measures: Lesion heat, swelling and pain evaluation (subjective; scoring scales not reported). Lameness assessment (subjective; scoring scale not provided); motion analysis system (objective; device not reported). Lesion CSA, circumference and transverse size. Colour Doppler signal: subjective; ordinal scale (0–5; 0 = no signal, 5 = strongly increased signal). Converted to a binary outcome: scores 0 or 1 = ‘no signal’; scores 2–5 = ‘increased signal’. Objective measures: Echogenicity percentage: objective, software-aided pixel count; calculated as lesion pixel intensity divided by pixel intensity of the non-injured portion of the ligament branch.
Main Findings (relevant to PICO question)	All horses: No heat, pain on palpation or swelling at lesion sites on day 1 post-surgery; mild to moderate heat, pain on palpation and swelling after week 1 (lunging at trot). No lameness at the walk (duration of the study). No skin burns or other adverse effects of HILT. Ultrasound: Ten ultrasound measurements taken (day 1–week 4, n = 12 horses; months 2–6, n = 6 horses). No significant difference between treatment and control for mean lesion CSA and mean lesion circumference (day 1–month 6). CSA enlargement at week 4 (week 4 CSA minus week 1 CSA) was significantly smaller (P = 0.01) in the treatment group compared to control. Additionally, circumference enlargement (week 4 circumference minus week 1 circumference) was significantly smaller in the treatment group compared to control (P = 0.016). This was depicted in Figure 5 of the paper; actual values not reported. Treatment group mean lesion transverse size was significantly smaller than control at month 2 (3.5 mm, standard deviation (SD) = 0.9 mm vs 4.3 mm, SD = 1.9 mm; P = 0.026) and month 3 (4.2 mm, SD = 1.0 vs 4.6 mm, SD = 1.9; P = 0.015). In six ultrasound measurements (week 4 to month 6), treatment and control mean echogenicity scores were significantly larger than their respective scores at week 3 (P < 0.001). Treatment group colour Doppler signal was significantly higher than control during the treatment period (day 1–week 4; P < 0.001). MRI: No significant differences between MRI scores of the three blinded evaluators. MRI results were consistent with the ultrasound and colour Doppler evaluations. In short- and long-term treatment groups, mean lesion CSA was significantly smaller (P = 0.002) and mean MRI signal was significantly lower (P = 0.006) than control.
Limitations	No follow-up beyond 6 months, no consideration of return to function (horses were euthanised). Selective reporting of significant differences of ultrasound measurements (treatment vs control) may result in reader interpretation bias. For example, in Table 2, 28/30 (93%) ultrasound measurements (treatment vs control) were not significant; however, there was no discussion of this. The healing properties of surgically induced suspensory branch lesions may differ from lesions occurring naturally due to injury. The authors do not state their reasons for choosing a surgical design rather than treating naturally-occurring lesions. No explanation was provided for having short- and long-term post-treatment evaluation groups. The length of time required to create the 48 surgically-induced lesions and the number of veterinary surgeons involved was not reported. The authors do not report the variance within the surgically-induced lesions to the parameters stated in the surgical protocol. The authors refer to Supplementary File 1 for details on ultrasound measurements; however, this file does not show echogenicity scores and the scores for CSA, transverse size and swelling do not match those in the paper. The description of statistical tests used to analyse ultrasound and MRI data was insufficient to appraise the suitability of the tests. It is unclear how many different MLMMs were used and how they differed. Confidence intervals are not reported. The lameness detection motion-analysis system was not specified. Power calculations were not reported. The study may be underpowered to detect differences in the measured outcomes; however, increasing the sample size would likely be unethical. The laser device software was custom to this study (details not provided); no information was provided regarding the testing or validity of the software. Mean and SD not reported for the ages of the 12 horses. The authors report no significant difference between the scores of the MRI evaluators but do not state the protocol for resolving scoring differences. The method of randomised assignment to treatment and control groups was not reported. The method of assignment to short- and long-term evaluation groups was not reported.

**Zielińska et al. (2020):** 

Population	Horses admitted to a single-centre veterinary hospital in Poland. Inclusion criteria: Clinical diagnosis of tendinopathy or desmopathy with at least two of; pain on palpation, limb swelling or lameness. Tendon or ligament injury confirmed by ultrasound. Injury not previously treated.
Sample size	Twenty-six horses with 29 tendon and ligament lesions. Group characteristics: Warmblood performance horses of both sexes (details not reported). Age = 5–24 years; mean = 11.5 years (SD not reported). Lesions: superficial digital flexor tendon (SDFT) (n = 12; 41%), deep digital flexor tendon (DDFT) (n = 8; 28%), suspensory ligament branch (SLB) (n = 6; 21%), suspensory ligament body (SL) (n = 3; 10%).
Intervention details	Treatment and control groups: Treatment (n = 23) and control (n = 6) group sizes were predetermined (details not reported). One horse had a SL injury in both forelimbs; the left limb was assigned to the treatment group; the right limb was assigned to the control group. Twenty-two tendon and ligament injuries were randomly assigned to the treatment group; the remaining five injuries were assigned to the control group. Clinical Assessment: Three clinical assessments, including ultrasound performed by the same veterinarian, measured pain response, degree of lameness, relative swelling, degree of lesion echogenicity and lesion size: before treatment (BT; day 0), during treatment (DT; days 13–15), after treatment (AT; days 38–40). Rehabilitation: All horses (treatment and control) received the same conservative rehabilitation (days 1–40): twice daily 20 minute walks on a hard surface followed by 20 minutes of cold-water hosing of the lesion. High-intensity laser therapy (HILT): Laser device: Astar Polaris HP S class 4 laser, maximum power 18 W, simultaneous 808 nm and 980 nm wavelengths. Horses in treatment group received 15 treatments (same veterinarian) beginning day 1. The time between successive groups of treatments was increased: four treatments (24 hours apart), four treatments (48 hours apart), four treatments (72 hours apart), three treatments (96 hours apart). The laser handpiece was held perpendicular to the skin (distance from the skin not reported). The laser device software calculated the duration and total energy dose of each treatment which varied according to lesion location (details not reported). Sedation was not required for HILT treatment. Ethics approval: Ethical Committee for Experiments on Animals, Wroclaw University, Poland.
Study design	Prospective, non-randomised, non-blinded, controlled clinical trial.
Outcome studied	Clinical measurements were taken three times: BT, DT and AT. Measurements taken: Pain on palpation: subjective ordinal scale (0–3; 0 = no pain, 3 = severe pain). Lameness assessment: subjective ordinal scale (0–3) using the American Association of Equine Practitioners (AAEP) scale; 0 = lameness not perceptible under any circumstances, 3 = lameness is consistently observable at a trot under all circumstances. Relative swelling: objective calculation of the percentage difference between the circumference of the injured limb at the lesion location and the circumference of the healthy limb at the same location. Lesion echogenicity (ultrasound): subjective ordinal scale (0–3; 0 = isoechoic, 3 = anechoic). Lesion percentage (ultrasound): subjective measurement, objective calculation (lesion percentage = lesion cross-sectional area / tendon or ligament cross-sectional area x 100).
Main Findings (relevant to PICO question)	Statistically significant differences reported between treatment and control at the end of HILT treatment for each outcome measured (pain, lameness, relative swelling, lesion echogenicity and lesion percentage). Pain (Pearson’s chi-square test): Treatment vs control: Treatment group had significantly lower AT pain scores than control (P = 0.023); 22/23 (95.7%) of treatment AT pain scores were (0 or 1) compared to 3/6 (50.0%) for control. Intra-group (BT vs AT): Treatment group AT pain scores significantly lower than BT scores (P < 0.001). Lameness (Pearson's chi-square test): Treatment vs control: Treatment group AT lameness scores were significantly lower than control (P = 0.04); 19/23 (82.6%) of treatment AT lameness scores were (0 or 1) compared to 3/6 (50.0%) for control. Intra-group (BT vs AT): Treatment group AT lameness scores significantly lower than BT lameness scores (P < 0.001). Mean relative swelling (one-way ANOVA, post-hoc Tukey test): Treatment vs control: Treatment group DT mean relative swelling significantly lower than control (4.2% vs 7.8%; P = 0.024). Treatment group AT mean relative swelling significantly lower than control (2.4% vs 6.8%; P = 0.008). Intra-group (BT vs AT): Treatment group AT mean relative swelling significantly lower than BT mean relative swelling (2.4% vs 6.4%; P < 0.001). Lesion echogenicity (Pearson's chi-square test): Treatment vs control: Treatment group AT echogenicity scores significantly lower than control (P = 0.001); 18/23 (78.3%) of treatment lesion echogenicity scores were (0 or 1) compared to 0/6 (0%) for control. Intra-group (BT vs AT): Treatment group AT lesion echogenicity scores significantly lower than BT lesion echogenicity scores (P < 0.001). Lesion percentage (Mann-Whitney U test): Treatment vs control: Treatment group AT mean lesion percentage significantly lower than control (15.7% vs 35.5%; P = 0.02). Intra-group (Friedman ANOVA): Treatment group: DT mean lesion percentage significantly lower than BT (20.7% vs 24.1%; P < 0.001). AT mean lesion percentage significantly lower than DT (15.7% vs 20.7%; P = 0.001). AT mean lesion percentage was significantly lower than BT (15.7% vs 24.1%; P < 0.001). Control group: AT mean lesion percentage significantly lower than BT (35.7% vs 39.7%; P = 0.04). Data variability: Control group interquartile ranges (IQRs) at BT, DT and AT were larger than those of the treatment group by factors of 4.0, 3.8 and 2.4, respectively. Treatment group maximum lesion percentage values at BT, DT and AT were 3.7, 3.7 and 4.4 times their respective third quartile (Q3) values. Control group maximum lesion percentage values were 1.1, 1.0 and 1.1 times their respective Q3 values. HILT safety: Horses treated with HILT did not experience skin burns, pain reactions or other adverse effects.
Limitations	Internal validity threatened by questionable methodology related to treatment / control group sizes, allocation randomisation, lack of blinding and statistical mistakes. Treatment and control groups: Treatment and control group sizes were predetermined, likely resulting in allocation bias (details not reported). Questionable method of randomisation to allocate lesions to treatment and control. Power calculations were not reported; this study may have been underpowered to detect clinically meaningful effects. The distribution of lesions by type in treatment and control groups was not reported; the number of SLB injuries in the treatment group is not known. Selection bias is likely as all horses were performance horses (breed and discipline not specified). The type and severity of lesions may not represent the general population. Standard deviation (SD) of horses' ages not reported. Methodology: All HILT treatments and clinical and ultrasound assessments were performed by the same unblinded veterinarian, increasing the risk of observer and confirmation bias. The majority of outcome measures were subjective. The laser device software determined the duration and energy dose of each HILT treatment (not reported). Information regarding the testing or validity of the laser device software was not reported. No post-treatment follow-up. The authors do not state how relative swelling percentage was calculated for the horse with SL lesions on both front limbs (no healthy limb to compare). Data analysis and reporting: Confidence intervals not reported. Risk of reader interpretation bias: Relative swelling: Table 2 in the paper shows the median, IQR and min-max values for percentage relative swelling, whilst Figure 2 shows boxplots of relative swelling percentage with mean, mean ± standard error (SE; boxplot body) and mean ± 1.96 x SE (boxplot whiskers). SE values not reported. Lesion percentage: Figure 5 in the paper shows boxplots (median, IQR, min-max) of treatment and control lesion percentage at periods BT, DT and AT. The authors do not discuss the large visual differences between treatment and control IQRs and maximum values (same y-axis scale), leaving interpretation to the reader.

## Appraisal, application and reflection

High-intensity lasers, also known as high-power, class IV and ND:YAG lasers, are characterised by an energy output greater than 500 mW and the ability to penetrate tissue to a depth of 5 to 15 cm (Ahmad et al., 2021). Human and veterinary medical applications of high-intensity laser therapy (HILT) include pain management and the treatment of tendinopathy, desmopathy and osteoarthritis (Zielińska et al., 2015; Fortuna, 2017; Ahmad et al., 2021; and Mongkolrat et al., 2021).

Two peer-reviewed controlled studies utilising HILT to treat equine tendon and ligament injuries were appraised; however, each study has design, methodological, statistical analysis and reporting issues that reduce reliability and external validity. Additionally, the lack of standardised protocols for HILT treatment of equine soft tissue injuries complicates comparisons of study methodologies (Fortuna, 2017). For example, each appraised study used a different commercial high-intensity laser device running custom software that determined the laser energy output and other treatment parameters based on lesion location. Importantly, the total energy administered during each HILT treatment was not reported, making it impossible to compare the treatment doses administered in the two studies. Further, the number of treatments and the time between treatments varied between the two studies; Zielińska et al. (2020) administered a total of 15 HILT treatments over 40 days (increasing time between successive treatments), whilst Pluim et al. (2020) administered one HILT treatment daily for 4 weeks.

The appraised studies differed in their implementation of conservative management. For example, in Zielińska et al. (2020), horses in both the treatment and control groups received twice-daily hand-walking on a hard surface (20 minutes) followed by cold-water hosing of the HILT-treated lesion (20 minutes). This protocol was administered for the 40 days of HILT treatment; however, it is unknown whether rehabilitation continued post-HILT. In the four-limb surgical model of Pluim et al. (2020), one diagonal limb pair (right front / left hind or left front / right hind) was randomly assigned to the treatment group, and the other diagonal limb pair was assigned to the control group. A progressive exercise programme commenced 2 weeks after the start of HILT, consisting of 20 minutes of hand-walking on a hard surface progressing to incrementally increasing trot and canter work. Dyson (2007; and 2018) reports that horses with suspensory ligament branch (SLB) injuries may require 9–18 months of conservative management depending on lesion severity before returning to primary function; however, neither appraised study achieved this guideline or considered return to function. Zielińska et al. (2020) did not follow their subjects post-HILT, whilst the horses in Pluim et al. (2020) study were euthanised 4 weeks post-HILT (short-term group) and after 6 months (long-term group).

Design decisions likely reduced the internal validity and potential clinical relevance of the two appraised studies. For example, the four-limb surgically-induced lesion model of Schramme et al. (2010), modified by Pluim et al. (2020) to create SLB lesions, may be of questionable validity. Estrada et al. (2014) found significant differences in the healing properties (tendinous and biochemical composition) between surgically-induced forelimb and hindlimb superficial digital flexor tendon (SDFT) lesions, suggesting additional research is needed to validate the four-limb model. Although the conclusions of Estrada et al. (2014) were based on surgically-induced tendon lesions, their findings may apply to surgically-induced ligament lesions and suggest the results of Pluim et al. (2020) be carefully considered. Additionally, the four-limb surgical model may present welfare concerns (Ribitsch et al., 2020). Neither appraised study reported power calculations; therefore, these studies may be underpowered to detect clinically meaningful effects of HILT treatment. Further, Zielińska et al. (2020) do not explain why the treatment (n = 23) and control (n = 6) group sizes were predetermined. Additionally, the allocation method employed by Zielińska et al. (2020) likely resulted in a non-randomised allocation of lesions between treatment and control, increasing the risk of selection bias (Christley & French, 2018). Zielińska et al. (2020) reported results that conflated lesion types, making it impossible to consider the effects of HILT on SLB injuries. Reporting results by lesion type may have increased the relevance of this study to the PICO question. Pluim et al. (2020) treated SLB lesions exclusively; however, their reported results may bias reader interpretation. For example, the differences between treatment and control mean transverse lesion size were significant in months 2 and 3; however, the authors fail to discuss the implications of the 28/30 (93%) other lesion measurements, including cross-sectional area (CSA), circumference and transverse size that were not significant.

Further, two reporting errors were found in Zielińska et al. (2020) Table 1, column ‘Degree of lesion echogenicity’. First, the chi-square test p-value for treatment vs control for AT lesion echogenicity was incorrectly reported as P = 0.70; the authors incorrectly concluded there was no significant difference between lesion echogenicity for treatment vs control (Type II error). Using the data provided in Table 1, the Knowledge Summary author calculated the chi-square test statistic and p-value using the ‘chisq.test’ function in R (version 4.1.1; R Core Team, 2021) and an online chi-square calculator (Preacher, 2001). The results from R and the online calculator were identical (P = 0.001), indicating a significant difference between treatment and control. All other reported p-values in Table 1 are correct. Second, for the control group, the AT frequency count and percentage for scale value = 1 was incorrectly reported as '0 (50.0)'; it should read '0 (0.0)'.

In summary, the two appraised studies provide insufficient evidence to show that horses treated with HILT and conservative management return to primary function sooner than horses treated with conservative management alone. The non-randomised controlled trial (Zielińska et al., 2020) showed significant effects of HILT; however, results were conflated by four different lesion types and return to function was not considered. The randomised controlled experimental trial (Pluim et al., 2020) selectively reported significant effects of HILT; however, overall results were inconclusive and return to function was not considered. Finally, there is limited understanding of the temperature effects of HILT on pigmented and non-pigmented equine skin; additional research in this area may have clinical relevance to HILT treatment of SLB injuries (Zielińska et al., 2021). Therefore, there is only weak evidence to show that HILT treatment of equine soft tissue injuries hastens lesion healing and return to primary function compared to conservative management alone.

## Methodology

**Search Strategy:** 

Databases searched and dates covered:	CAB Abstracts on OVID platform (1973–2022 Week 35) PubMed on NCBI platform (1910–2022 Week 35) Web of Science Core Collection (1900–2022) Embase on OVID platform (1980–2022 Week 35)
Search strategy:	CAB Abstracts exp horses/ OR horse* OR equine* ((high* adj4 laser) OR (class* adj4 laser) OR HILT OR ND:YAG OR "ND YAG") tend* OR ligament* OR desmopath* 1 AND 2 AND 3 **PubMed**: (horse* OR equine*) AND laser AND (high* OR "high intensity" OR "high power" OR class* OR ND:YAG OR "ND YAG") AND (tend* OR ligament* OR desmopath*) Note: A non-truncated version of the above PubMed search string (all search words spelled out) returned only four papers; therefore, the PubMed search was conducted using the truncated version of the search string. **Web of Science**: (horse* OR equine*)** **AND** **laser AND (high* OR "high intensity" OR "high power" OR class* OR ND:YAG OR "ND YAG")** **AND (tend* OR ligament* OR desmopath*) Embase (horse* OR equine*) AND laser AND (high* OR "high intensity" OR "high power" OR class* OR ND:YAG OR "ND YAG") AND (tend* OR ligament* OR desmopath*)
Dates searches performed:	06 Sep 2022

**Exclusion / Inclusion Criteria:** The PICO question considers equine suspensory ligament branch injuries treated with high-intensity laser therapy (HILT). The initial literature search (restricted to equine ligament injuries) returned only four papers; consequently, the search was broadened to include studies that treated equine tendon or ligament injuries with HILT.

Exclusion:	Non-English language, non-equine studies, studies using high-intensity laser therapy to treat other than equine tendon / ligament injuries, conference proceedings, non-systematic reviews, papers published before 1985, abstract-only papers, studies without a control group, papers lacking sufficient details to appraise.
Inclusion:	Controlled studies in which high-intensity laser therapy was used to treat equine desmopathy or tendinopathy, systematic reviews.

**Search Outcome:** 

Database	Number of results	Excluded – Not directly related to PICO question	Excluded – No control group	Excluded – Insufficient details to appraise	Excluded – Papers published before 1985	Excluded –Non-systematic review or conference proceedings	Excluded – Abstract only	Excluded – Not English language	Total relevant papers
CAB Abstracts	11	2	2	0	0	2	0	3	2
PubMed	28	22	2	0	2	0	0	0	2
Web of Science	31	19	2	0	0	3	1	4	2
Embase	8	2	2	1	0	1	0	0	2
Total papers when duplicates removed									2

## Acknowledgements

The author would like to thank Dr Louise Buckley and Maureen O’Mara PhD for their guidance and encouragement.

## ORCID

James Rushing: https://orcid.org/0000-0002-7451-5707

## Conflict of Interest

The author declares no conflicts of interest.
